# Integrating cognition in the laboratory with cognition in the real world: the time cognition takes, task fidelity, and finding tasks when they are mixed together

**DOI:** 10.3389/fpsyg.2023.1137698

**Published:** 2023-08-24

**Authors:** Thomas H. Carr, Catherine M. Arrington, Susan M. Fitzpatrick

**Affiliations:** ^1^Program in Cognition and Cognitive Neuroscience, Department of Psychology, Michigan State University, East Lansing, MI, United States; ^2^Department of Psychology, Lehigh University, Bethlehem, PA, United States; ^3^LSRT Associates, St. Louis, MO, United States; ^4^James S. McDonnell Foundation, St. Louis, MO, United States

**Keywords:** cognitive studies, mental chronometry, ecological validity, timescales of cognition, task fidelity, speed-accuracy tradeoff, multitasking, task signatures

## Abstract

It is now possible for real-life activities, unfolding over their natural range of temporal and spatial scales, to become the primary targets of cognitive studies. Movement toward this type of research will require an integrated methodological approach currently uncommon in the field. When executed hand in hand with thorough and ecologically valid empirical description, properly developed laboratory tasks can serve as model systems to capture the essentials of a targeted real-life activity. When integrated together, data from these two kinds of studies can facilitate causal analysis and modeling of the mental and neural processes that govern that activity, enabling a fuller account than either method can provide on its own. The resulting account, situated in the activity’s natural environmental, social, and motivational context, can then enable effective and efficient development of interventions to support and improve the activity as it actually unfolds in real time. We believe that such an integrated multi-level research program should be common rather than rare and is necessary to achieve scientifically and societally important goals. The time is right to finally abandon the boundaries that separate the laboratory from the outside world.

## Introduction

Exhortations to expand the repertoire of methodologies beyond experimental cognitive psychology are not new, but pursuing them successfully is now more achievable than ever. Admirable efforts in this direction can be found in many domains of cognition, including learning and memory (e.g., Brown et al., 2014; Roediger and Karpicke, 2018; Rawson and Dunlosky, 2022), visual cognition and spatial navigation (e.g., Denis et al., 2014; Zhao et al., 2021; Kelly et al., 2022; Shayman et al., 2022), mathematical cognition (e.g., Rittle-Johnson, 2019; Murphy et al., 2022), mind wandering (Smallwood and Schooler, 2006, 2015; Kane et al., 2007, 2017a,b), and clinical investigation of psychological and neurologic disorders and injuries (e.g., Marcotte and Grant, 2010; Weizenbaum et al., 2020). A particularly compelling account of a cognitive-developmental psychologist’s journey from laboratory to real world and back comes from Adolph (2020), who closes by saying “Researchers in any field must remind themselves that participants’ abilities in structured lab tasks do not necessarily reflect participants’ actual behaviors outside the lab. And the best way to ensure ecological validity in structured lab tasks is to start with a rich description of real-world behavior.”

We begin our own examination of these issues from the point of view of mental chronometry—the study of human performance in real time. We argue for a multilevel analysis in which single research programs incorporate cognitive-computational, neurobiological, and real-life ecological studies into integrated and well-coordinated empirical and theoretical characterizations.

### On every trial, please give me the fastest correct response you can

In the cognitive laboratory, the time people take to get something done has been harnessed and formalized into a set of paradigms broadly referred to as mental chronometry, using the speed and accuracy with which a task is performed under intentionally varied conditions to infer the nature, organization, and operation of the mental processes by which the task is carried out (e.g., Sternberg, 1969; Dosher, 1976; Posner, 1978, 2005; McClelland, 1979; Wickelgren et al., 1980; Townsend and Ashby, 1983; Logan and Cowan, 1984; Meyer et al., 1988; Abrams and Balota, 1991; Carr, 2005; Jensen, 2006; Van Zandt and Townsend, 2006; Townsend et al., 2015; Wascher et al., 2022). To get data amenable to such inferencing, task performers are commonly given an instruction something like this: “On every trial, please give me the fastest correct response you can.”

### Response time in cognitive psychology

Why do cognitive psychologists think that “the fastest correct response” is so important? Getting participants to follow such an instruction was fundamental to cognitive psychology’s growth into a serious science capable of drawing reliable inferences about mental processes from measures of speed and accuracy in laboratory settings (Keele, 1973; Posner, 1973, 1978; Lachman et al., 1979; Townsend and Ashby, 1983; Gardner, 1985). To elucidate the hidden cognitive operations that govern observable actions, experimental cognitive psychology and more recently experimental cognitive neuroscience have used data collected from precisely specified and usually heavily crafted goal-directed activities set for laboratory volunteers to carry out according to particular instructions. Such a goal-directed activity is called a “task.” If the participant follows the task’s instructions and the laboratory equipment measures the participant’s performance in reliable and reproduceable ways, then cognitive analyses can be conducted on the data. The goals of cognitive analyses are to infer the computational and functional architectures that support the experimental task and the mental operations carried out in those architectures to achieve the task’s execution. To succeed, cognitive scientists need to be confident that the measurements taken during the participant’s performance are capturing the targeted task, completed in full, and only the targeted task. If other things are mixed in, cognitive scientists need to be able to identify them and separate out their contributions to the data. Mental chronometry wants measures of the task, the whole task, and nothing but the task. Hence the instruction, intended to constrain performance toward that end.

Compelling cognitive analyses emerged gradually, from a series of major conceptual, analytic, and methodological advances that were powerful stimulants for further advances: additive factors logic, isolable subsystems logic, processes in cascade, speed-accuracy and response-deadline logics, random-walk and race modeling using reaction-time data, connectionist modeling of computational architectures and operations, statistical modeling of the independent contributions of multiple factors to brain activity as seen in fMRI and other neural measures, neural network modeling of nervous-system architectures and operations. By now, empirical data from rigorous experimentation has contributed to systematic theoretical analysis, quantitative modeling, and simulation modeling of task performances, at both the level of mental computations and the level of brain activity. Behavioral data underlying cognitive psychology’s task modeling have stood up reasonably well to the scrutiny of the “replicability crisis.” Neural data, in particular data obtained *in vivo* using fMRI, EEG, and MEG, have had a somewhat rockier time, suffering some serious criticisms regarding both interpretability and reliability, but have nevertheless been able to support a solid and growing body of cognitive-neuroscientific modeling done within the constraints of laboratory-based experimental tasks.

### So where is the problem?

As a result of focusing on the desiderata of experimental control and precision of task analysis, we now have a mature body of laboratory-generated cognitive science findings that map to the computational, neurophysiological, and neuroanatomical levels of description. But do the findings obtained in laboratory tasks under rigorous experimental control—and typically very short time scales—explain human cognition as it unfolds over the time scales and complex motivational topographies of real life activities? Sometimes the answer is yes, but only when something like the integrated approach extolled in our preface is being followed.

It is important to keep in mind that laboratory tasks are deliberately artificial, heavily crafted in service of particular experimental goals, and such tasks might or might not actually capture the mental processes underlying any given real-world activity. The risks of moving from the environment and timescales of the typical laboratory experiment to those of everyday life are compounded through the use of terms that have both scientific and colloquial meanings. Consider the potential for semantic creep that can occur in using an experimental participant’s ability or inability to inhibit a pre-potent motor response as measured in the Stop Signal Reaction Time Task or the Go/No-Go Task as a basis for explaining why an individual is “impulsive” in real-world decisions and actions (for reviews of the evidence and its complexity, see Bari and Robbins, 2013; Smith et al., 2014; Esteves et al., 2021). It was very exciting to find correlations between performance in those laboratory tasks and the degree to which task participants had previously been identified as overly impulsive or hyperactive. However, it must be kept in mind that a very specific experimental cognitive task designed to test a specific cognitive model somehow became a diagnostic test and perhaps an explanatory model for a very complicated family of human behaviors. The inferential leap from performance in the SSRT or the Go/No-Go Task to the complexity of real life is long and complicated. If there is a linkage between performance in a stopping or response-withholding task and the likelihood that one will demonstrate a lack of impulse control and ultimately engage in behaviors that are considered risky or socially undesirable, then what is it?

There are multiple considerations that must be addressed in establishing such a linkage. One issue, of course, is the fidelity between the goals, task demands, and underlying processing streams in which the two phenomena are occurring. The processing that fails to inhibit a prepotent response and the processing that produces an impulsive decision or action could be identical to one another, or they could be partially overlapping, or they could be useful analogies of one another but sharing no actual processing content, or they could be completely unrelated except by some third factor that makes them correlated. After all, the laboratory tasks seem so very different from deciding on a whim to turn left in traffic or take up a friend on a dare, and even further from the choice to engage in sky diving for the thrill of it. This means that observing a correlation between one of the laboratory tasks and the real-life constructs of impulse control or thrill seeking is necessary but not sufficient for determining the utility of the laboratory task as a model system for understanding the real-world phenomenon. As Heuer (1988, p. 405) so eloquently put it, “Generalization of experimental results seems to require a high similarity of the experimental situation to a reference situation, while generalization of theoretical statements requires invariance of the system, that is, invariance of functionally defined components and their interrelations.”

The second issue is the range of temporal scales over which the causal factors and processing streams in the two situations unfold. Inhibition as deployed and measured in the SSRT must necessarily occur in a brief burst within the course of a task that lasts only a second or two from start to finish, and its bursts must be under the control of the participant and repeatable on demand every few seconds for a couple of dozen to a hundred or so repetitions over the course of an experiment that might last half an hour to an hour. Whether what might be called “impulsivity” in real-life activities—lacking the ability to control one’s behavior in response to cues from within oneself or from the environment—(1) results from failures of control processes that (2) occur in brief bursts that (3) are under the control of the actor and (4) unfold over time courses that bear some resemblance to the (5) temporal parameters, and (6) repetition requirements of the cognitive laboratory is a very real set of questions. And again, these are questions that are not answered by the mere existence of a correlation between a measure of Stop Signal or Go/No-Go reaction time taken in the laboratory and a measure of how impulsively someone might be judged to behave in a real-life situation.

Some progress toward building a more integrative methodology that could move research forward can be seen in recent work combining laboratory tasks using behavioral and neural measures, psychological scales asking for self-ratings, and experience-sampling techniques for assessing real-life daily activity and experience. One example is Hur and colleagues’ (2022) hybrid analysis of anxiety. fMRI measurements of frontocortical and amygdala activation during tasks involving either threat anticipation or viewing of negative-affect-inducing facial images were correlated with rating-scale measures of neuroticism and trait anxiety, and both were used to predict reactions to everyday positive and negative events obtained through experience sampling. Participants received text messages several times a day containing a questionnaire in which they rated dimensions of positive and negative affect being experienced at that time, and whether a negative event or a positive event had been experienced during the past hour. The main results were first that the threat-anticipation and negative facial image tasks produced their standardly obtained in-laboratory findings of increased reports of stress, anxiety, and negative emotion, accompanied by frontocortical activation, during threat anticipation, and amygdala activation while seeing negative facial images. The interesting advance was made in observing the relations between these expected findings and the reports obtained from experience sampling. Greater frontocortical activation when anticipating laboratory-induced threat predicted less emotional distress following negative experiences in real-life activity. Given that the observed imaging changes occurred in frontocortical regions known to be involved in a number of other tasks that require inhibitory cognitive control, the neural findings suggested to Hur and colleagues a regulatory dampening of distress in real-life experience managed by neural processing mechanisms that could be tapped and observed during specific laboratory tasks, cognitive as well as emotional. Thus the hybrid methodology directly relating short-timeframe laboratory observations to longer-timeframe measures of real-life activity narrowed the field of possibilities for how either the laboratory data or the real-world data might be interpreted on their own.

### Complex multi-level tasks whose components operate on different timescales

Beyond differences between the timescales and contexts of laboratory and real-world task activities, there are important domains of human activity that are themselves complexly organized with component processes operating over quite different timescales despite interacting and depending on one another. An example is the development and deployment of language communication skills as described by Piazza et al. (2021, p. 459):

When adults and young children communicate, they exchange information across milliseconds, seconds, and minutes. Statistics of these exchanges accumulate through diverse interactions across hours, days, and months and have long-lasting consequences for children’s cognition. Children are tasked not only with integrating communicative input across the set of shorter timescales from milliseconds to minutes (e.g., connecting related words into meaningful sentences and narratives), but also with aggregating experiences across many interactions. (Gogate and Hollich, 2010; McMurray, 2016; Altmann, 2017)

Piazza and colleagues go on to advocate for an approach to modeling multi-timescale processes capable of capturing this complexity. The idea is that statistical summary processes operate at each of the multiple levels of the processing hierarchies that develop and implement language comprehension and production: sounds, symbols, gestures, words, syntactic units, meanings, narrative organizations. These levels must be coupled and synchronized in order for comprehension to be triggered by sensory input or for an idea to be successfully turned into speech, writing, or sign. Quantitative tools have become available for characterizing processes of statistical summary, discovering neural processing hierarchies, and describing coupling across two or more streams of neural processing. Piazza and colleagues discuss how these tools might be applied together to achieve psycholinguistic analysis in naturalistic settings in which language communication involves multiple interlocutors at multiple levels of skill and may include, alternate with, or be interrupted by other kinds of activities. Such research would require detailed behavioral observations with concomitant neural data (for example from wireless-enabled EEG nets) collected in real-world settings over multiple extended sessions—a challenge indeed, but one worth pursuing.

The potential that lies in integrating analyses across multiple timeframes can be seen in a recent study of patterns of infant exposure to objects and their names by Clerkin and Smith (2022). Mealtime observations revealed two different timescales of object-name experience. From meal to meal, objects from a variety of categories were frequently present, allowing their visual representations to become well-established in memory. However, an object in any given category was only rarely named. Within each meal, objects were often named, but any given object-name pairing occurred in only a few mealtime episodes. A spoon might be used but not named while a bowl might be used and named, and a fork might be neither used nor named during any one meal. Nevertheless, the frequency with which each object-category name occurred was enough to support associative learning according to analyses of complimentary cortical and hippocampal learning processes characterized by McClelland et al. (1995), Merhav et al. (2015), Hebscher et al. (2019), and Ellis et al. (2021).

As an example of the type of multi-level, multi-method approach we are advocating, Clerkin and Smith (2022) is limited because it did not itself include neural evidence that could speak directly to the involvement of the cortical associative mechanisms hypothesized to be doing the work in learning from these patterns of experience operating at different timescales, but such evidence was appropriately imported and mapped onto the observational and statistical evidence they did collect. A similar approach was taken toward joint infant-caregiver attention and its impact on vocabulary development by Abney et al. (2017), demonstrating the versatility of multi-timescale analyses across different targets of investigation.

### The fastest response you can give? sometimes slower cognition might be better cognition and in the real world it might be very common

Regardless of its utility for laboratory-based modeling efforts, in the real world a fast correct response may not always produce the best outcome for the situation, and sometimes even if it would be best, the performer may not be able to give it. Thus, the theoretical achievements of laboratory-based mental chronometry may cover only a small territory in the universe of human cognitive activity.

Spencer (2016), a teacher, wrote eloquently on why he believes that slow and even sporadic thinking can be at least as important, and often more satisfying, than coming up with “the fastest correct response you can give.” He acknowledged that trying to be speedy yet accurate is sometimes necessary. But he went on to suggest that there is a profound place for deeper thinking that takes more time. Divergent and creative engagement with real-world problems that may take weeks or months to solve involves a more slowly unfolding and more reflective kind of cognition than the rapid-fire analysis that is the strong suit of computers and AI chatbots, is often emphasized in school, and is so often asked for in the laboratory.

Our first reaction is that Spencer is right. Sometimes the fastest correct response is no more than adequate, if that. It may be correct in a pedestrian sense but lack the creativity and panache to be a real accomplishment. Our second reaction is that Spencer is wrong, for society and also for scientific studies. This is because *when corrected for “quality”*—if we can figure out how to judge quality with sufficient validity and reliability in whatever the situation is that we are looking at—we still want “the fastest correct response” *to the problem being solved in the way it is being solved*. We need such data if we are to understand the processes of solution, model them effectively, and perhaps determine how to improve them or make them more efficient (happening faster in a busy and demanding world but still special in the qualitatively desirable way). Therefore the challenge is how to get an empirical picture of cognition that allows for slow, reflective, and creative thinking yet captures just that thinking for the purposes of theory building, modeling, and intervention. Before focusing on the task, the whole task, and nothing but the task, we need the truth, the whole truth, and nothing but the truth about what “the task” really is when it is a part of real life rather than abstracted and condensed for laboratory purposes.

### Tasks and task performance in the real world

Embracing a science of behavior in all its richness requires a willingness to depart from the clean elegance of laboratory task performance, despite the power it provides for cognitive analysis. What might be called cognitive-ethological or cognitive-anthropological methods for real-world observation of “cognition in the wild” have been developed to high levels of complexity and quality (see, e.g., Marcotte and Grant, 2010; Kirsh, 2011, 2013; Hutchins, 2012). By themselves, however, they are not sufficient to get a complete multi-level picture of what cognition is, how it works, and what it accomplishes. Thus, we are not advocating for abandonment of experimental tasks in favor of research based solely on real-world observation. Instead, we advocate for integrating laboratory tasks as model systems for understanding well-specified cognitive performances into larger research programs that include real-world observation at a macro-level as well as neural and kinematic measurements at micro-levels.

Implementing such programs raises a series of questions about timescales, schedules of unfolding performance, and the impact of mixing task performances together in the string of activities that makes up a human being’s rather messy real-world life. Developing rigorous ways to ask these questions in well-orchestrated programs of ecologically relevant yet analytically rigorous research would greatly advantage the field’s ability to meet the challenges of ecological fidelity and understanding that are hard to achieve in laboratory work alone.

Inspired by John Spencer, we might investigate when is it better to go slowly than to give the fastest response you can, even if that response is nominally correct, and how is it better. Some progress has been made on this question in laboratory studies of speed-accuracy tradeoff, error monitoring, and pursuit of gains versus aversion to losses. The literature is filled with evidence that people can be induced to go faster or slower, to emphasize speed or to emphasize accuracy, and to value gaining rewards or avoiding losses in a specific task under controlled conditions. Furthermore, we know that there are conditions that force or create slowing down, that make slow performance a necessary consequence rather than an option under experimental or personal control. Most people slow down as they get older because nerve transduction speed declines. Stroke and traumatic brain injury patients, during and sometimes after recovery, go more slowly out of necessity because nervous system circuits supporting performance have been damaged.

But what happens outside the specific constraints imposed in cognitive experiments or by the exigencies of aging, damage, and disease? We can see from accident reports, recollections of students taking tests, and the like that people sometimes choose to go faster than conditions and abilities will support, but do we have a clear picture of when and why that happens and, when it does, what the real-world speed-accuracy tradeoff functions actually look like? One effort to find out examined 29 data sets representing a wide range of cognitive tasks, tests, and standardized assessments administered in real-world settings (Domingue et al., 2022). Variations in response time did not necessarily predict variations in accuracy—the calculated speed-accuracy tradeoff functions varied widely. But that study does not tell us why. Within each data set there were individual differences, often showing that poorer performers showed greater variation of accuracy with speed, but even those larger variations did not always show the kind of speed-accuracy function observed so standardly in the laboratory. More work on this issue can and should be done.

We can also ask the question a different way: Do people commonly go more slowly than they could and if so, what does that look like and why do people do it? There are multiple possibilities:

(1) Perhaps they want extra time to rethink, to check their work—mental or physical—before taking the next step or declaring the task completed. We know from laboratory studies that novices in a new task need and usually prefer to go slowly, prepare carefully, and double-check, sometimes on a step-by-step basis, if they are to avoid mistakes and improve at the task. Performance speeds up with practice, increasing expertise, and increasing confidence. We also know from laboratory studies that experts in at least some kinds of activities do not need to go slowly to be successful unless the task situation is very unusual or presents serious challenges not dealt with before, and performance can even be degraded by slowing down in a well-mastered task (Beilock et al., 2004a,b, 2008; Burns, 2004). However, most of us, even experts, slow down and increase step-by-step attentional control in pursuit of accuracy and to avoid error when we feel pressure to perform at our very best and there are consequences for failure. Laboratory investigations show that such a strategy can work out well for novices but backfire for experts (Beilock and Carr, 2001; Beilock and Gray, 2007). How well do these principles hold in the wide range of real-world activities and real-world contingencies with which people deal in their ongoing daily duties? And where does the difference between *needing* to slow down and merely *wanting* to slow down come into play? What are the causes and consequences of one versus the other?
(2) Perhaps people are not always performing the task? This could happen because task performers get tired and need a rest or they get bored and seek a mental diversion or their performance simply falls victim to a bout of mind wandering (Smallwood and Schooler, 2006). In particular, mind wandering decoupled from awareness (which has been called “zoning out”) results in more rapid but less accurate performance in a simple continuous performance task, but when individuals become aware of a mind wandering episode, even intend one (“tuning out” rather than “zoning out”), they actively slow performance (Smallwood et al., 2007). Considerable effort is currently going into finding ways of identifying mind wandering both in the laboratory and in real-world environments such as school classrooms, and in developing interventions to control it (see, e.g., Christoff et al., 2009; Szpunar, 2017; Liu et al., 2020; Kane et al., 2021).
(3) Perhaps people are always performing *some* task, but not always the *same* task. That is, they are choosing to bounce around from one goal pursuit to another. How often and under what circumstances people select to move among tasks is studied as voluntary or self-organized task-switching in the laboratory (Arrington et al., 2014; Mittelstädt et al., 2018). Another possibility is that people get switched, interrupted, or diverted by a call to do another task or service another activity, and they must make the change whether they want to or not. There is quite a bit known about cued or externally instigated task switching, but again in the laboratory (Grange and Houghton, 2014; Schneider, 2015). Whether driven by some external interruption or selected voluntarily, switching between tasks adds time to performance in the lab, typically on the order of tens or hundreds of milliseconds per switch even between tasks that themselves take only a second or two when done in isolation, and can increase the probability of errors as well.

These laboratory findings on voluntary and externally instigated task switching tell us what happens when a small set of well-characterized tasks is posed in a limited experimental environment. How much of what we know about task switching translates into accurate understanding, either empirical or theoretical, when people go to work at their jobs or set out to do a few errands on an ordinary day? Does switching among task activities result in the same degree of slowing or loss of accuracy when there are many fewer constraints on exactly what people might choose to do next from their intended task list, or be told to do next by the boss, and with the added possibility that unpredictable surprises might call for their attention in either kind of setting? After all, many individuals in real-world settings express a preference for multitasking and assume that it will speed overall performance if tasks are interleaved, leading to greater productivity (Sanbonmatsu et al., 2013; Sanbonmatsu et al., 2016). Are they wrong in this assumption, as laboratory research would suggest, or are there types of tasks or circumstances of performance in which they are right?

### Can we tell such possibilities apart? looking for task signatures in the laboratory and the real world

What are the signatures of these various performance profiles—can we distinguish them from one another and identify which ones are happening in any given period of performance? If we can distinguish them, can we then identify the consequences of each kind of profile—what is gained and what is lost, and at what benefits and costs? And can we make these determinations in real-life activity as well as in the laboratory, so that we can successfully integrate between them? Analytic techniques are available that we believe can be applied to finding in a real-life task’s activity the cognitive processes captured in a laboratory task, and vice versa. Serious complications arise, however, when tasks are stretched out in time, interrupted, or multitasked. While tractable in the laboratory, can we deal with these complications in real life? There we may be looking for a laboratory task’s activity in a family of real-life tasks, or for a family of real-life tasks in a mixture of real-life families. These are formidable challenges to take on. What tools are currently available for trying to tackle them? Here is a sampling that seems to us to be promising:

(1) Application of event-structure analysis for identifying possible real-life / laboratory-task isomorphisms (Radvansky and Zacks, 2014). As an example, see Zacks and Dennis (2020, Event Cognition in the Wild, https://www.jsmf.org/grants/2020-1143/), a project funded by the James S. McDonnell Foundation. Using an electronic data collection system called Unforgettable, Zacks and Dennis are applying event-structure analysis to experiential data containing times, locations, people involved, accelerometry-based actions, images, sounds, language, weather, and emotional states and reactions, all self-recorded during daily life by some 2,700 participants. With such data, comparisons of contents and temporal properties of laboratory events and tasks can be compared with examples of “the same” events and tasks as they unfold in the real lives of people out in the world.
(2) Application of Multivoxel Pattern Analysis (MVPA) techniques to matrices of observational and chronometric data for discovering task signatures and searching for them in real-life activity. The challenge in this approach would lie in identifying an appropriate array of quantifiable properties of the environment, the performers’ bodies, nervous systems, and phenomenological reports, and the performers’ actions whose measurement would provide the matrix of time series on which MVPA analysis would operate. These measurable properties would correspond to the voxels from which time series of metabolic measures are taken when MVPA is applied to brain activity. Suppose the targeted task, such as “rinsing a plate” to take an example from daily life, is either carried out in isolated fashion as a lab task or embedded in the course of a variety of different other events, such as those completed when washing dishes or doing something less related such as cooking, which might vary in their coherence and their relatedness to one another or to the target event. Does that target event stay the same (it could be plugged into any sequence just as it is, something like a routine from a programming library that can simply be inserted into a larger program) or does it vary substantially from instance to instance or from one performance context to another (analogous, perhaps, to co-articulation effects in speech production—see, e.g., Browman and Goldstein, 1989, 1992, as suggested by Jeffrey Zacks, personal communication, April 28, 2022). An analytical approach that determines whether or not a task performance is the same in the lab as it is in its real-life versions, and how much variability in each of its properties exists from one instance of performance to another, would build a key bridge between research on cognitive processes at multiple temporal scales. A major obstacle to be overcome would be finding an action corpus rich enough in terms of event types and accompanying data streams to support the approach’s development. An existing data base worth trying (again suggested by Jeffrey Zacks, personal communication, April 28, 2022) might be https://psyarxiv.com/r5tju/.
(3) Application of common recording techniques that provide points of connection and comparison across laboratory and real-world tasks. Electrophysiology and neuroimaging approaches that allow for engagement in everyday activities, such as Ambulatory EEG and functional Near Infrared Spectroscopy (fNIRS) for classifying brain states, can support inferences about cognitive processes from neural processing and discovery of task signatures common across laboratory tasks and real-life activities. An example of such a combined approach is a program of research relating cognitive control and rule learning during controlled laboratory tasks to student engagement with intelligent tutoring systems (ITSs; Unal et al., 2020; Howell-Munson et al., 2023). Commonly used in both classrooms and remote learning, ITSs can provide precise timing through collection of extensive log data, which can be supplemented by experimental tools designed to capture and log behaviors in online environments. Combined with machine learning techniques, fNIRS applied during both ITS and controlled laboratory tasks has promise for linking common cognitive mechanisms across tasks that occur on different time scales with materials of different complexity (Howell-Munson et al., 2021). Additionally, assessment techniques now commonly used to tap cognitive processes in real-world settings, such as think aloud protocols, have long been used for analytic purposes in the laboratory (e.g., Chi et al., 1989; Ericsson and Simon, 1993; Yang, 2003) and can be combined with behavioral data and fNIRS data (Howell-Munson et al., 2023). Thus, progress can be made through a convergent approach that brings measurement techniques from real-life and laboratory domains to bear together in targeting common cognitive mechanisms across domains.
(4) Application of Ihlen and Beatrix’s (2010) interaction-dominant dynamical systems analysis for identifying continuities and discontinuities in performance time series that signal interactions between tasks and changes from one task to another. This quantitative tool might complement the MVPA-based analysis proposed above, and perhaps the two could be used in concert. In turn, MVPA and dynamical system analysis might be coordinated with time-series techniques specifically aimed at identifying mixtures of interdependent activities that change over time, either microgenetically or as a function of cognitive development. An introduction to such techniques can be found in Xu et al. (2020).
(5) Application of traditional context-comparison and individual-differences approaches in novel and analytic ways. An example is Altmann et al.’s (2022) work on the relation between laboratory experimenter-controlled administration and on-line self-administration of a complex laboratory task involving working memory maintenance, sequential performance, and recovery from disruption. How do characteristics of the task performance and its relations to individual differences stay the same or change when moving from the laboratory to the considerably less constrained context of online self-administration? Are there conclusions that stand across experimental environments and other conclusions that must change because the results change? Another example of such an investigation is a master’s thesis by Katsumata (2022) on whether choking under pressure can be induced as a reliable phenomenon in an online self-administered version of a pressure-induction paradigm that has succeeded in the laboratory. While these two examples do not stray far from laboratory-task mental chronometry, the environments in which online self-administration operates are much more varied, much less controlled, and much more susceptible than the laboratory situation to naturally-occurring interruptions, multitasking, mind wandering, loafing, and even cheating. That is, they introduce a dose of real life’s possibilities into the staid atmosphere of laboratory research.
(6) Even when using standardly crafted laboratory tasks, more can be learned about how those tasks do and do not map onto the real-world experience that participants bring into the laboratory. Polman and Maglio (2022) propose adding to each experiment a questionnaire assessing familiarity with the context, situation, and/or stimuli, calling this assessment a “reality check.” Their examples come from decision-making scenarios investigating supposedly well-established phenomena such as transaction utility, sunk-cost effects, and delay discounting. In each of seven experiments a measure of familiarity or interest in the situation and/or the stimuli moderated the results. Only one of the phenomena—delay discounting—appeared at all levels of the reality-check variable, and even then, its magnitude varied substantially with participant experience and interest in the task scenario. While some experimental tasks might be more easily or sensibly amenable to a “reality check” than others, applying this notion where possible would increase the ecological interpretability of laboratory experimentation.

### Back to the future

At present, then, in the laboratory, cognitive processes are usually investigated by constructing a task intended to expose a particular choice, decision, or goal-directed action to experimental control, manipulation, and measurement, or to isolate a particular component process of the performance so that it can be manipulated and its operating characteristics measured. These tasks do not usually take long to perform. A single instance of perceptual judgment, word recognition, or targeted reaching might be over in three-quarters of a second or less. Some performances might require a few seconds or a few minutes, such as studying a word list, solving a reasoning problem, working a complex math problem, taking a golf putt on a laboratory green, or completing a circuit in a driving simulator, but these are extended performance requirements by laboratory standards. Trial timelines, temporal task parameters, and measures of performance properties are commonly expressed in milliseconds or seconds, occasionally in minutes. While repetition is important in the laboratory—participants typically engage in a task multiple times during an experimental session—the entire session might last as little as 20 min and rarely exceeds an hour and a half. Two hours would be quite a long stretch in a cognitive laboratory.

When studies require learning, retention intervals between encoding opportunity and memory test are often within the same single session. If a more extended retention interval is interrogated, it might be a day, a few days, or a week. Longer-term retentions—months, years—do get studied, but such investigations are a rarity, only a small percentage of the many studies of what is called without irony “long term memory.” One example of rigorously conducted research with experimental control over both the conditions of original learning and subsequent testing is Kolers’ (1976) report of the persistence for a year of text-specific familiarity effects in the speed and accuracy of reading mirror-image text. A striking focus on retention intervals as long as 50 years characterizes Bahrick’s well-known studies of how much former Ohio Wesleyan University students could remember of their Spanish lessons, the layout of the small city in which their school is located, and their classmates’ names and faces (see Bahrick, 1979; Bahrick et al., 2013). But while memory testing was done with a high degree of experimental rigor, including careful attempts to document retention intervals and opportunities for relearning since leaving college, none of these studies of naturalistic memory maintenance had any control over the original learning experiences.

There are research areas not dominated by small timescales. In acquired expertise and in some domains of developmental psychology, longer bouts of time on task and more repetition over longer periods of time are considered. For example, the so-called “10,000 hour rule” in attaining expert status in a complex skill (Ericcson et al., 1993) comes to mind—whether or not it is correct (Hambrick et al., 2014; Macnamara et al., 2014). Substantial retention intervals over which to observe the impact of an earlier experience are considered a necessity for some studies, as in attempts to document the longevity of gains made during early intervention (e.g., Lee et al., 1990; Isaacs, 2008; Welsh et al., 2020) or interactions between aptitude and training in the development of extraordinary success in math, science, music, and other domains (e.g., Simonton, 1988, 1999; Lubinski and Benbow, 2006). Classroom studies of school achievement have shown that performance on tests of classroom learning deteriorates over a summer vacation, so that assessments of math or social studies are lower at the start of the next school year than they were at the end of the previous year. Again, however, such studies are rarely able to exert experimental control over experiences of learning, though some studies are able to establish treatment versus non-treatment comparisons or utilize “natural experiments,” for example involving different laws in comparable communities or change in law from one time to the next in a single community (e.g., Card and Kreuger, 1994) or arbitrary cutoff dates for entrance into a societal activity such as formal schooling (Morrison et al., 2005; Connor et al., 2009). “Big data” analyses of public data sets apply econometric techniques to establish longitudinal and generational changes in education, income, health, economic mobility and other important life features (e.g., Chetty et al., 2014, 2016, 2020a,b; Carr et al., 2020; Carr and Wiemers, 2022), but are generally unable to establish the sorts of intraindividual processing dynamics that produce the perceptions, memories, thoughts, decisions, and actions underlying such long-term trends that would constitute a cognitive analysis.

Thus it appears that there are tradeoffs in which the unfolding of real-world versions of the parent phenomena happening on their natural timescales can be studied, but at present that is usually done at a cost to precise cognitive analysis, whereas experimental control and precision of analysis are gained by modeling phenomena in laboratory tasks, but at the cost of losing fidelity to the processing streams and timescales of cognition in its natural environment. Reducing, eliminating, and eventually reversing this tradeoff is a paramount challenge for cognitive science. We believe that the field can gain ecological fidelity while maintaining—or even increasing—rigor and precision of analysis. Making common what is now a rare kind of methodology, one that integrates observation, laboratory tasks taking a variety of controlled measures, cognitive-computational and neurobiological modeling, development of intervention, and evaluation of the intervention’s impact in a single extended line of investigation, is a goal worth pursuing. To achieve this goal, we need to increase both the ecological interpretability of analytic laboratory tasks and the laboratory-analytic compatibility of real-world observations.

The James S. McDonnell Foundation made support for work pursuing these needs a priority in a call for “Opportunity Award” proposals issued in 2020 from its Understanding Human Cognition Program. That program, recently concluded, funded 28 Opportunity Awards representing a wide range of takes on how one might go about getting this sort of research underway. Descriptions of all the funded projects can be found at https://www.jsmf.org/grants/legacy/opportunity/index.php. We believe they represent steps in the right direction.

### Concluding with a success story: achieving task fidelity, temporal fidelity, and ecological relevance in a rigorous analysis of distracted driving

In 2001 Strayer and Johnston published “Driven to Distraction: Dual-Task Studies of Simulated Driving and Conversing on a Cellular Phone.” This was the first report from what became an ongoing research program that has redefined what imposing laboratory-level analytic rigor, pursuing task and temporal fidelity in comparing laboratory to real-world methods and results, and achieving ecological relevance can look like in high-quality cognitive research. The findings have furthered theoretical understanding of human cognition, illuminated the workings of an important domain of human-machine interaction, and inspired the formulation of new social and legal policy to increase public safety.

Strayer and Johnston’s (2001) study implemented a laboratory-task analog of driving in a version of the pursuit tracking task using a joystick to keep a cursor on a dot moving at varying speeds across a visual display. To this “driving” task was added a laboratory analog for events that require quick braking: a go/no-go task in which a light occasionally occurred in the field of view. The light could be red or green. If red, the “driver” had to push a finger button mounted on the joystick. If green the “driver” continued “driving”—that is, tracking. After 7 min of instruction and practice, the “driver” performed this “driving and braking” task by itself in two 7.5-min bouts, separated by a 15-min bout in which “driving and braking” was combined with one of several potentially distracting language-processing tasks: (1) conversing with a confederate on a hand-held cell phone (the joystick controlled with one hand, the phone held in the other), (2) conversing with a confederate on a hands-free cell phone, (3) listening to a radio broadcast of the “driver’s” choosing, (4) listening to a book of the experimenter’s choosing, with a memory test afterward so data could be included only from “drivers” who processed the book passage sufficiently to answer simple questions about its content, (5) repeating each of a series of words heard on a hands-held cell phone, and (6) generating a new word starting with the last letter of each of a series of words heard on the hands-held cell phone. Results showed that relative to the single-task baseline, three conditions showed decreased pursuit-tracking accuracy, greater numbers of missed red lights in the go/no-go task, and longer go/no-go reaction times when red lights were detected: generating new words from the words heard on the cell phone, conversing on the hand-held phone, and conversing on the hands-free phone. Surprising to many people—including legislators considering new rules for cell phone use while driving—was that conversing on the hands-free phone produced just as much interference as the hand-held phone. Strayer and Johnson concluded that “These data are consistent with an attention-based interpretation in which the disruptive effects of cell-phone conversations on driving are due primarily to the diversion of attention from driving to the phone conversation itself.”

From this start, which relied on analog laboratory tasks performed for short periods of time by minimally trained participants, Strayer and colleagues moved toward greater task fidelity, temporal fidelity, and ecological relevance. Soon experiments were conducted in driving simulators, and measures of vehicle control such as deviation from the center of the driving lane were added to the original response-time and accuracy measures required for mental chronometry. By the 2020’s physiological measures had been added as well, such as heart rate and heart-rate variability. At present, most data are collected during extended bouts of driving done by licensed drivers in real cars on real roads. Laboratory tasks are still employed, but for two kinds of specific purposes. The first is taking measurements or making comparisons that would be too dangerous to do in the field such as Strayer et al.’s (2006) comparison of cell phone distraction to drunk driving, or literally impossible, such as using fMRI to do blood-flow-based brain mapping while driving. FMRI data have been collected during simulated driving by Schweizer et al. (2013), using an immersive virtual reality system in an MR scanner. The second purpose for which laboratory methodology has been maintained is in testing specific theoretical hypotheses about processing, such as Strayer et al.’s (2021) comparison of the time needed to recover from secondary-task interruption during pursuit tracking versus simulated driving. This comparison examined the relative importance of cleansing working memory of the interrupting task (important regardless of the primary task) versus rebuilding situational awareness (which was thought to be crucial for driving but minimal in pursuit tracking).

The output of Strayer’s research program has been a multi-level characterization of a human-machine interaction with world-wide relevance. The societal impact of this work has already been enormous, over and above its theoretical and human-factors contributions. Of course, no research program is ever perfect. Young (2015, 2018) has reanalyzed some of Strayer’s findings, compared them to real-world driving data in new ways, and identified compensatory strategies by which drivers try to deal with the risk of dividing attention during driving. We see the careful crafting and ongoing development of Strayer’s program and the serious follow-ups it has elicited as a success story in the pursuit of analytic rigor, task and temporal fidelity, and ecological relevance. It is a model to be emulated.

## Author contributions

All authors listed have made a substantial, direct, and intellectual contribution to the work and approved it for publication.

## Conflict of interest

SF was employed by LSRT Associates.

The remaining authors declare that the research was conducted in the absence of any commercial or financial relationships that could be construed as a potential conflict of interest.

## Publisher’s note

All claims expressed in this article are solely those of the authors and do not necessarily represent those of their affiliated organizations, or those of the publisher, the editors and the reviewers. Any product that may be evaluated in this article, or claim that may be made by its manufacturer, is not guaranteed or endorsed by the publisher.
